# Comparison of Rabies Cases Received by the Shomal Pasteur Institute in Northern Iran: A 2-Year Study

**DOI:** 10.1155/2023/3492601

**Published:** 2023-02-25

**Authors:** Saeid Kavoosian, Ramezan Behzadi, Mohsen Asouri, Ali Asghar Ahmadi, Mehrab Nasirikenari, Alireza Salehi

**Affiliations:** ^1^North Research Center, Pasteur Institute of Iran, Amol, Iran; ^2^Islamic Azad University, Science and Research Branch, Tehran, Iran; ^3^Zoonoses Research Center, Pasteur Institute of Iran, Amol, Iran; ^4^Department of Pathology, Babol Branch, Islamic Azad University, Babol, Iran

## Abstract

The rabies virus, which belongs to the genus *Lyssavirus*, the family *Rhabdoviridae*, is the causative agent of rabies, a contagious, deadly, and progressive neurological infection. This illness is commonly distributed worldwide and affects all warm-blooded animals. Regarding the zoonotic aspects of rabies, the prevalence of rabies was investigated in this study. Over 2 years, 188 samples were examined via the direct fluorescent antibody test (DFAT) and mouse inoculation test (MIT) techniques by using brain tissue samples. Our findings showed that 73.94% of samples were rabies positive. The highest number of samples belonged to cows and dogs, respectively. The positivity rate in cows was 71.88%, followed by dogs with a 57.78% infection rate. These findings suggested that despite the heavy monitoring protocols in Iran, rabies is still a prevalent disease, and it is advised that vaccinations and screening programs should be carried out more frequently with heavier observation.

## 1. Introduction

According to the International Committee on Taxonomy of Viruses (ICTV), rabies is caused by lyssaviruses, including the rabies virus and the Australian bat lyssavirus. It spreads when an infected animal (host) scratches or bites another animal or human [1]. Rabies is a viral disease with inflammation of the brain in humans and most mammals. Early symptoms include fever and tingling at the site of exposure in most animals including humans [2]. There are other symptoms such as violent movements, uncontrolled excitement, fear of water, inability to move parts of the body, confusion, and loss of consciousness. When symptoms appear, prognosis is mostly death [3]. The time between encountering the virus and the beginning of symptoms is usually one to three months but may vary from less than one week to more than one year [1]. The length of this golden time depends on how far the virus must travel along the peripheral nerves to reach the central nervous system (CNS) [4]. Infected saliva can also transmit the virus if saliva makes contact with mucus, for example, by contacting the eyes, mouth, or nose. Regarding rabies, dogs are the most famous host worldwide [1]. More than 99% of rabies-infected cases were caused by dogs by simply getting bitten [5]. However, in the USA, bat bites are the most common source of rabies infections in humans and less than 5% of cases are from dogs [1, 5]. Also, it should be noted that rodents are unlikely to be infected with rabies [5]. In northern Iran, most cows are mixed-breed including mixed Simmental and mixed Holstein [6]. They are the main source of meat, dairy products, and consequently income [7]. Meanwhile, dogs are not mostly kept by farmers, and they are also mixed-breed. This lack of ownership leads to a brutal competition for limited food resources and turns dogs into aggressive animals in this region.

Unfortunately, rabies can only be diagnosed after symptoms appear. However, animal control and vaccination programs have reduced the risk of catching the disease from dogs in some regions. Thus, immunizing people before they are exposed is recommended for those at high risk, including those who work with bats or spend prolonged periods in areas of the world where rabies is common. In people who have been exposed to rabies, the rabies vaccine and sometimes rabies immunoglobulin are effective in preventing the acute disease if the person receives the treatment before the start of rabies symptoms [1]. Washing the bitten and scratched areas for 15 minutes with soap and water, povidone iodine, or any antiviral detergent may reduce the number of viral particles and may be effective in preventing transmission [1, 8]. In 2016, only fourteen people survived the rabies infection after symptoms were presented [9, 10]. Rabies is estimated to cause 59000 human deaths annually in over 150 countries, with 95% of cases occurring in Africa and Asia [1]. To be more specific, according to the WHO, 31000 human deaths occurred in Asia, with nearly 20000 of them concentrated in India [11]. About 40% of deaths occur in children under the age of 15 [2]. Rabies is present in more than 150 countries and on all continents except for Antarctica [1]. More than 3 billion people live in regions of the world where rabies likely occurs [1]. Several countries, including Australia and Japan, as well as much of Western Europe, do not have rabies among dogs [12, 13]. Also, many islands do not have rabies at all [13], so it is classified as a neglected tropical disease [14].

The Rabies Laboratory of the Pasteur Institute of Iran has been known as the national reference for rabies diagnosis since the beginning of rabies diagnostic tests in Iran. In addition, according to the national protocol, the major reference for diagnosing all suspected rabies samples in humans and domestic and wild animals is the Pasteur Institute of Iran. For example, an earlier study of rabies infection was conducted in 2015 [15]. As was reported, the positivity of suspected cases was 59.5% among 116 samples, and it was mostly observed among dogs. Accordingly, this study was conducted to assay the prevalence of rabies among different animal species between 2016 and 2018 in order to evaluate the changes in the rabies infection rate.

This study was conducted at the Shomal Pasteur Institute in the north of Iran, which is one of the four Pasteur research centers in Iran. This institute is mainly responsible for rabies monitoring across the whole country since its establishment, and the Shomal center covers the four provinces of Ardabil, Gilan, Golestan, and Mazandaran. Therefore, in this study, all suspicious samples from these provinces were considered.

## 2. Materials and Methods

### 2.1. Sample Collection Protocols

**Figure 1:**
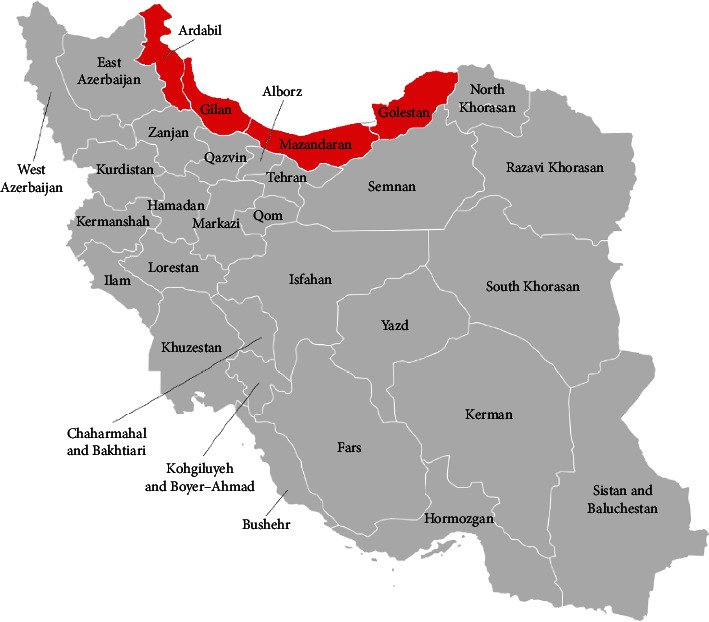
Investigated provinces in this research. The red parts are regions that were under observation, and samples were collected.

This experimental study was based on samples, which were received from four provinces of Iran (Ardabil, Gilan, Golestan, and Mazandaran) by the North Research Center of the Pasteur Institute of Iran in the city of Amol from March 2016 to March 2018 (Figure 1).

Based on the protocol, the bitten domestic animals were quarantined for 15 days, and in case of rabies symptoms, they were euthanized. The brain samples were collected from suspected animals and packed in a vial containing 50% glycerin and 50% phosphate buffer saline (PBS) [15]. In the case of no symptom detection, the suspected animal was kept in quarantine for another 15 days, and if no rabies symptoms appeared, it was returned to its original place of living.

Regarding wild animals, if they were trapped by veterinarians or environmental officers while showing bitten or any suspicious signs, they were first quarantined according to the same procedure, and if symptoms appeared, they were euthanized. The brain sample was sent to the laboratory. These wild animals act as a link (carrier) between wildlife and domestic animals. Furthermore, they can be the main carriers of the virus from its reservoir (jackal) to other animals.

### 2.2. Direct Fluorescent Antibody Test (DFAT)

Smear preparation started by opening the received vials. A small piece (20–50 mg) of the brain was picked up with a tip and placed on a wooden spatula. An imprint of the sample was made by pressing a glass slide on the piece of the brain over the spatula. The smear was fixed in high-grade cold acetone. 0.1 ml of clarified anti-rabies nucleocapsid conjugate (Bio-Rad, France) was placed on the sample, and the sample was incubated at 37°C for 30 minutes. Following incubation, the smear was rinsed twice with distilled water after several minutes of being stored in PBS. The stained samples were then analyzed under a fluorescent microscope (Motic AE30-31, Spain) for the presence of Negri bodies [15–17].

### 2.3. Mouse Inoculation Test (MIT)

While a positive DFAT result indicates an infection, a negative result does not rule out the possibility of infection. Therefore, after microscopic observation of the negative samples, the brain tissues were prepared and the mouse inoculation test (MIT) was performed. The MIT was conducted by adding a clarified supernatant of a homogenate of the brain material in an isotonic buffered solution containing antibiotics (10 ml of brain suspension with 50 *μ*l of penicillin and 20 *μ*l of streptomycin). Then, the skull of ten mice, 3–4 weeks old (12–14 grams), was injected with the suspension of 50 *μ*l of the brain sample. The mice were anesthetized by ether before inoculation. For intracerebral inoculation, sterilized needles (27 and 26 gauges) were used. Next, the mice were observed daily for 28 days. Any deaths occurring during the first 4 days were regarded as nonspecific, and every dead mouse after the 4th day was considered positive, and the brain was obtained and sent for the DFAT examination [17, 18].

### 2.4. Statistical Method

The collected data were analyzed using SPSS software (IBM Corp. Released 2019. IBM SPSS Statistics for Windows, Version 26.0. Armonk, NY: IBM Corp), and the chi-square test [19] was employed to compare the number of suspected and positive samples. *p* < 0.05 was considered a significant difference [20]. Moreover, a *p* value was estimated down to 0.0001 for further confidence levels [21, 22].

## 3. Results

In this study, 188 specimens were tested for rabies infection. Out of the 188 suspected wildlife specimens, 137 cases of rabies were confirmed as positive. Among these, the cow's brain tissue samples had the highest percentage, followed by dogs with 34 specimens, and the lowest proportion belonged to the horses, foxes, and camels with only one sample (Table 1). Jackals, wolves, foxes, horses, and camels had the most positive rabies rate in terms of animal species as 100% of the samples from these animals were positive.

Comparing the samples regarding the provinces, the highest rate was in Gilan province with 86 cases, followed by Ardabil with 50 cases (Table 2). Also, cows had the highest number of infected cases among all animals with 92 cases (66.19%), followed by dogs, sheep, and goats, respectively (Table 1).

## 4. Discussion

This study investigated the received samples from four provinces in the north of Iran, which consisted of ten different species. The samples were examined carefully and evaluated via the DFAT technique. After analyzing the data, it turned out that 72.87% of cases were rabies positive. Generally, animal bites can cause serious health problems [23], and exposure to stray dogs was reported as the major cause of human bites [24].

Most of the studied regions in this research are covered by forests and mountains, which have made it difficult to monitor rabies. In addition, all four provinces share a border with the neighboring countries of Iran, which makes it a lot harder to control animal reservoirs inside the country and decreases the possibility of controlling animals that travel between the borders. In order to control rabies and reduce human casualties, programs such as education of people and students, vaccination, and serum therapy of individuals and animals are among the most important, which, despite spending a lot of money, were not greatly successful in controlling the disease.

An earlier study was also conducted on rabies epidemiology in northern Iran [25]. As can be seen in Table 3, the overall rate of rabies cases has decreased in all four provinces after almost 15 years. However, the number of rabies-infected cases in cows, which are the most important and frequent animal, was reduced in the current study compared to that in the previous one. The reason is likely related to a better vaccination protocol during these years. In addition, an earlier study [25] showed a significantly higher infection rate in this area, but fortunately, the overall positivity of cases was massively decreased in this study.

**Figure 2:**
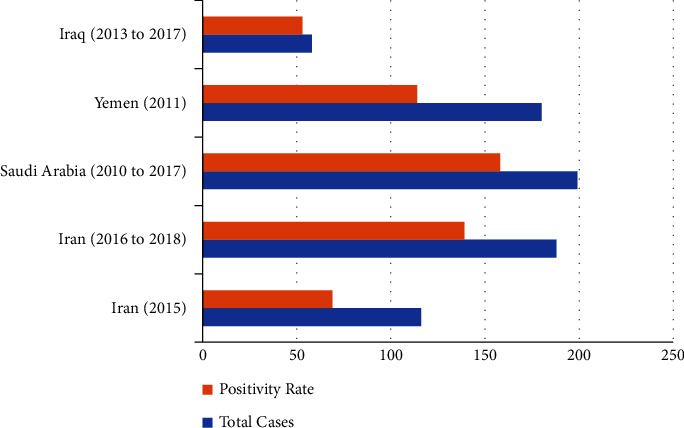
Comparison of the number of rabies-suspected cases and the positivity of them between Iran and other countries during the same time [15, 31, 33–35].

Also, there are certain areas, where animals potentially could carry the virus [26, 27]. The result of this study was in line with those of previous studies in neighboring countries, which indicated an almost similar condition of virus dispersion in those areas [28–31]. As an example, it was observed in an earlier study in Saudi Arabia that there are 158 (79.4%) positive cases among 199 suspected animals [31], while this infection rate in Iran was slightly lower (Figure 2). According to our findings, the largest sample proportion belonged to cows, of which 71.88% were rabies positive. Also, more than half of the rabies cases occurred in Gilan province. This may be due to the forests in that area and poor vaccination status, of which the latter requires quick and effective treatment protocols. Given the economic value of a cow, its vaccination can prevent its mortality chance, which directly affects economic and livestock markets. Moreover, the results showed that dogs in the northern provinces of Iran were substantially infected with rabies. In addition, Mazandaran province had the highest number of rabies cases in dogs, with 52% of them being rabies positive, while Gilan province had the highest positive rate with 64%. Regarding the behavior of the dog and how aggressive it can get, the animal can act as a vector for the virus. Dogs can share this disease with a wide range of humans [32]. They can also be the reason why the positivity rate of the cow was high since most of the dairy units in the area are still traditionally operated. To be brief, since stray dogs are the most common carrier and cows are the easiest target for being attacked by those dogs, there is no surprise that most of the suspected cases belonged to cows. Also, cows are the main source of meat in some areas, and they are likely to make contact with stray dogs more than other species. However, there could be a higher number of infected cows, but some bitten animals were kept in villages or jungles, which were never found or examined, and these kinds of cases were not similarly included in most of these kinds of studies.

These results are consistently similar to those of the previous studies [36–41] (Table 3). However, it should be noticed that in a study by Rahpeyma et al. [15], the data included all provinces, while this study focused on the four northern provinces. This is probably the reason for an increased rate of positive cases (Table 3). Regarding wild animals including jackals, wolves, and foxes, all samples were positive for rabies. This high prevalence can be a sign of the irrefutable contamination of wildlife animals, as well as their role in the survival of the virus in nature. The findings are similar to the findings of Wolfe et al. [42, 43]. The 100% prevalence of rabies in any animal can be a serious warning about the need to complete antirabies prophylactic therapy after getting bitten by these animals [44].

According to an earlier epidemiological study, rabies is an endemic infection in Iran, and it is a critical zoonotic disease. As was seen previously, the main transmitter of rabies is wolves. According to our findings, the incidences of rabies in animals and following humans are increasing annually. For example, more than 130000 people received postexposure prophylaxis in 2006 [45]. Also, data suggested that the majority of human exposures were due to dog biting. However, investigations showed that, in many cases, wolves were responsible for rabies transmission to humans [45]. In this study, wolves were shown to be a critical threat with a 100% infection rate.

Nevertheless, we can hope for the eradication of rabies when either the virus is removed from animal reservoirs or animal bites are managed to be inhibited. According to our findings, a total of 96.28% of the samples sent to the laboratory by veterinary centers were of domestic animals and 77.35% of them were of cows, sheep, and goats. The major reason for this high number of farm animals among the suspected cases is the high amount of interaction between these animals and wild carriers. The other possible reason is that government supports livestock farmers to eliminate infected animals by paying compensation for preventing the entry of contaminated meat into the country's food cycle. Also, it should be noted that one of the reasons behind the low number of wild animal samples is the difficulty of tracking wild animals in remote areas.

The wildlife spectrum in the northern provinces of Iran is diverse. Each region has some different carnivores. The infection of these animals with the rabies virus indicates the need for studying the density of these animals on the one hand and the need for immunization of these animals on the other hand. Countries are often attributed to low prioritization, epidemiological and operational constraints, and insufficient financial resources. Unfortunately, this was due to the lack of referral to health centers after exposure to an infected dog, which requires more information on prevention after an anomalous reminder. The disease is also prevalent in the countries of the region [46]. Finally, it must be noted that the only limitation was the lack of access to the wild biters in jungles and outskirts.

## 5. Conclusion

The findings of this study showed that Gilan, Ardebil, Mazandaran, and Golestan provinces had the highest rates of contamination, respectively. In the northern provinces, employing the control programs and the elimination of rabies can result in identifying the main hotspots. The findings of this study are supported by the fact that despite the wide range of annual measures against prevention, there is rabies currently. Unfortunately, the disease is still endemic with a high prevalence in Iran and is still one of the most important health challenges. It is suggested that control programs, such as vaccinations, especially in cows, and dogs, should be carried out with prophylaxis and increased community awareness of the disease to reduce and eliminate rabies disease.

## Figures and Tables

**Table 1:** Distribution of suspected and positive (infected) samples in Iran between 2016 and 2018.

	Animals	Suspected cases	Positive (infected) cases	Positivity percentage (%)
Domestic animals^a,c^	Cow	128	92	71.88
	Dog	34	26	76.47
	Sheep	8	6	75
	Cat	5	2	40
	Goat	4	4	100
	Camel	1	1	100
	Horse	1	1	100

Wild animals^d^	Wolf	3	3	100
	Jackal	3	3	100
	Fox	1	1	100

	Total^f^	188^e^	139^b^	73.94^g^

^a^Significant difference between the number of infected domestic animals with Pearson chi-square value 36.614 was *p* < 0.006. ^b^Significant difference between the number of infected domestic and wild animals with Pearson chi-square value 50.267 was *p* < 0.012. ^c^Significant difference between the numbers of suspected domestic animals with Pearson chi-square value 25.798 was *p* < 0.011. ^d^Significant difference between the numbers of suspected wild animals with Pearson chi-square value 5.000 was *p* < 0.025. ^e^Significant difference between the numbers of suspected animals with Pearson chi-square value 35.187 was *p* < 0.0001. ^f^Significant difference between the total number of domestic animals with Pearson chi-square value 49.471 was *p* < 0.0001. ^g^Significant difference between the total number of infected and suspected animals with Pearson chi-square value 69.362 was *p* < 0.0001.

**Table 2:** Comparison of suspected cases (percentage of positive cases) in different provinces of Iran. Each value presents the number of suspected cases (with the infection rate percentage).

Provinces	Dog (%)	Cow (%)	Sheep (%)	Wolf (%)	Cat (%)	Camel (%)	Jackal (%)	Goat (%)	Horse (%)	Fox (%)	Total cases (% of infected)
Gilan	—	**81 (75%)**	1 (100%)	—	1 (0%)	—	**2 (100%)**	1 (100%)	—	—	**86 (45.74%)**
Ardabil	11 (64%)	24 (77%)	**4 (50%)**	**3 (100)**	**3 (33%)**	**1 (100%)**	—	**2 (100%)**	**1 (100%)**	**1 (100%)**	50 (26.6%)
Mazandaran	**23 (52%)**	10 (90%)	3 (67%)	—	1 (100%)	—	1 (100%)	1 (100%)	—	—	39 (20.74%)
Golestan	—	13 (76.9%)	—	—	—	—	—	—	—	—	13 (6.91%)
Total infected cases (% among all infected cases)	34 (24.46%)	128 (66.19%)	8 (5.76%)	3 (2.16%)	5 (3.6%)	1 (0.72%)	3 (2.16%)	4 (2.88%)	1 (0.72%)	1 (0.72%)	188 (72.87%)

Bold values represent the highest number in each column.

**Table 3:** Comparison of the findings of previously conducted studies in Iran with those of the current study.

Conducted research	Results	Comparison to the current findings
Esfandiari et al. [25]	100% rate in Golestan	Golestan reduced to 6.91%
	90.5% rate in Gilan	Gilan reduced to 45.74%
	85.3% rate in Ardabil	Ardabil reduced to 26.6%
	64.3% rate in Mazandaran	Mazandaran reduced to 20.74%

Rahpeyma et al. [15]	50% of dogs	Dogs increased to 76.47%
	85% of cows	Cows decreased to 71.88%
	43% of sheep	Sheep increased to 75%
	55% of wolves	Wolves increased to 100%
	100% of jackals	Jackals remained at 100%
	60% of foxes	Foxes increased to 100%
	75% of goats	Goats increased to 100%
	75% of donkeys (horses)	Horses increased to 100%
	Overall 59.5%	Overall increased to 73.94%

## Data Availability

Data are available on reasonable request from the corresponding author.
